# Association between number of hyperbaric oxygen therapy sessions and neurocognitive outcomes of acute carbon monoxide poisoning

**DOI:** 10.3389/fmed.2023.1127978

**Published:** 2023-02-14

**Authors:** Je Seop Lee, Yong Sung Cha, Jihye Lim

**Affiliations:** ^1^Department of Emergency Medicine, Yonsei University Wonju College of Medicine, Wonju, Republic of Korea; ^2^Research Institute of Hyperbaric Medicine and Science, Yonsei University Wonju College of Medicine, Wonju, Republic of Korea; ^3^Department of Biostatistics and Center of Biomedical Data Science, Yonsei University Wonju College of Medicine, Wonju, Republic of Korea

**Keywords:** carbon monoxide poisoning, cognitive dysfunction, hyperbaric oxygen therapy, prognosis, session

## Abstract

**Background:**

Hyperbaric oxygen therapy (HBO_2_) is recommended for symptomatic patients within 24 h of carbon monoxide (CO) poisoning. Currently, there is no consensus on the number of HBO_2_ sessions within 24 h after arrival at the hospital. Therefore, we evaluated differences in the therapeutic effects according to the number of HBO_2_ sessions in acute CO poisoning.

**Methods:**

This cohort study included data collected from our CO poisoning registry and prospective cohorts between January 2006 and August 2021 in a single academic medical center in South Korea. Based on the number of HBO_2_ sessions performed within 24 h, we classified patients into one- and multiple- (two or three) session groups. We also compared mild (non-invasive mechanical ventilation) and severe (invasive mechanical ventilation) groups. CO-related neurocognitive outcomes were measured using the Global Deterioration Scale (GDS; stages: 1–7) combined with neurological impairment at 1 month after poisoning. We classified GDS stages as favorable (1–3 stages) and poor (4–7 stages) neurocognitive outcomes. Patients belonging to a favorable group based on GDS assessment, but with observable neurological impairment, were assigned to the poor outcome group. Propensity score matching (PSM) was performed to adjust for age, sex, and related variables to identify statistical differences between groups.

**Results:**

We analyzed the data of 537 patients between ages 16 and 70 years treated with HBO_2_. After PSM, there was no significant difference in neurocognitive outcomes at 1 month among the two groups of patients (*p* = 0.869). Furthermore, there were no significant differences in neurocognitive outcomes between invasive mechanical ventilation and non-invasive mechanical ventilation patients in the three groups (*p* = 0.389 and *p* = 0.295).

**Conclusion:**

There were no significant differences in the reduction of poor neurocognitive outcomes according to the number of HBO_2_ sessions implemented within 24 h of CO exposure.

## 1. Introduction

In the United States, approximately 50,000 patients with carbon monoxide (CO) poisoning are admitted to the emergency departments (EDs) of hospitals annually, with 1,500 deaths reported annually (1–3). Neurocognitive sequelae develop in 25–50% of acute CO poisoning survivors (4). Neurocognitive sequelae, including gait and motor disturbances, cognitive dysfunction, hearing loss and vestibular abnormalities, dementia, depression, and psychosis, are variable and can be permanent (5).

Of the six published randomized controlled trials (RCTs) that evaluated the effect of hyperbaric oxygen therapy (HBO_2_) in acute CO poisoning (4, 6–10), the most well-designed double-blind RCT by Weaver et al. satisfied all the elements of the Consolidated Standards of Reporting Trials (CONSORT) (4), and compared normobaric oxygen therapy (NBO_2_) with three sessions of HBO_2_ that were implemented within 24 h after arrival at the hospital. They reported that in patients arriving at the hospital within 24 h after CO poisoning, the intervention significantly reduced the incidence of cognitive sequelae at 6 weeks and 12 months post-treatment. In another RCT, Thom et al. (8) observed patients with mild to moderate CO poisoning and compared a group that received one session of HBO_2_ within 6 h with a group that received atmospheric oxygen. They found that HBO_2_ reduced the frequency of delayed neuropsychological sequelae (DNS). Therefore, one or three sessions of HBO_2_ performed within 24 h of CO poisoning may be a reasonable recommendation for patients with symptomatic CO poisoning.

Although there is no current consensus on the number of HBO_2_ sessions within 24 h, three sessions are recommended, according to the best-performing RCT study by Weaver et al. (4), by the Undersea and Hyperbaric Medical Society (1). Although Weaver et al. (4) performed three HBO_2_ sessions within 24 h after hospital arrival, they did not compare the number of HBO_2_ sessions implemented within 24 h. Additionally, Thom et al. (8) showed that one HBO_2_ session alone might be effective in acute CO poisoning. Therefore, we do not know how many sessions of HBO_2_ treatment within 24 h after ED arrival are most effective in preventing neurocognitive outcomes in acute CO poisoning. Therefore, we evaluated the difference in therapeutic effect according to the number of HBO_2_ sessions [one vs. multiple (two or three)] in patients who received HBO_2_ therapy within 24 h after ED arrival in CO poisoning.

## 2. Methods

### 2.1. Study population

Study data were derived from a cohort from a single tertiary academic hospital in South Korea. Our study included patients with acute CO poisoning enrolled from January 2006 to August 2021 at Wonju Severance Christian Hospital. Patients were followed up until February 2022. Since January 2006, a CO poisoning registry has been used to prospectively collect consecutive patient data in our hospital. After August 2020, data were collected prospectively with informed consent for our “CARE CO cohort” (ClinicalTrials.gov identifier: NCT04490317). We included adults (excluding those under 16 and over 70 years of age due to age-related changes in neurocognitive function) with acute CO poisoning who were administered HBO_2_ therapy within 24 h after rescue from CO exposure, without additional HBO_2_ sessions after 24 h after ED arrival. Additionally, the following patients were excluded: (1) those with a history of stroke or neurocognitive disorder, (2) those with previous CO poisoning, (3) those with serious illness, such as advanced cancer, which can affect the prognosis, (4) those undergoing other specific treatment (therapeutic hypothermia or steroids) because their effects on prognosis is not known, (5) those lacking follow-up for neurocognitive outcome at 1 month, and (6) those with insufficient data for important variables including HBO_2_ sessions.

### 2.2. Hyperbaric oxygen therapy

Our institute has three monoplace and one multiplace chambers as well as four hyperbaric physicians and two trained nurses. Acute CO poisoning is diagnosed according to patient history and a carboxyhemoglobin (CO-Hb) level of >5% (>10% for heavy smokers). We treated patients with 100% oxygen therapy through a facemask with a reservoir bag. Patients with any loss of consciousness intervals, neurocognitive symptoms or signs, cardiovascular dysfunction, elevated cardiac enzymes, ischemic electrocardiogram changes, severe acidosis, or CO-Hb ≥25% were treated with HBO_2_ (1). During the first HBO_2_ session, initial compression was performed with 2.8 atmospheres absolute (ATA) for 45 min, followed by 2.0 ATA for 60 min (8). The number of HBO_2_ sessions was determined by the physician who treated the patient and the resources of the HBO_2_ center because there is no consensus on the number of HBO_2_ sessions within 24 h. The second and third sessions were performed with 2.0 ATA for 90 min, all within 24 h after ED arrival.

### 2.3. Study variables and definitions

We evaluated the following clinical variables in patients with CO poisoning: age, sex, the intentionality of CO poisoning, CO source (charcoal, gas and oil, or fire), drug co-ingestion, Glasgow coma scale (GCS) at the time of rescue or ED arrival, comorbidities (diabetes mellitus, hypertension, cardiovascular disorder, or psychiatric disease), alcohol co-ingestion, current smoking status, any interval of loss of consciousness, shock, seizure, duration of CO exposure, times from rescue to the first HBO_2_, and the number of HBO_2_ sessions within 24 h after ED arrival. Laboratory variables included CO-Hb, bicarbonate, lactate, creatinine, creatine kinase, and troponin I (see the Supplementary methods for further details regarding variable definitions).

We classified the patients into one- and multiple (two- or three-) session groups based on the number of HBO_2_ sessions performed within 24 h. Moreover, mildly and severely poisoned patients were defined as those not requiring and requiring invasive mechanical ventilation, respectively (11).

CO-related neurocognitive outcomes were measured using the Global Deterioration Scale (GDS; stages: 1–7) (12) combined with neurological impairment (Supplementary methods; Supplementary Table 1) at 1 month (4–6 weeks) after CO poisoning. For patients at GDS stages 1–7, impairment ranged from none (stage 1) to loss of motor skills (including walking) and loss of all language skills, except for inaudible unintelligible sounds (stage 7). Patients who died during CO poisoning treatment within 1 month were assigned stage 7, which was the most severe stage. We classified GDS stages as favorable (1–3 stages) and poor (4–7 stages) neurocognitive outcomes (13). Patients belonging to a favorable group based on GDS assessment, but with observable neurological impairment, were assigned to the poor neurocognitive outcome group. We investigated the GDS stage combined with neurological impairment at 1 month post-CO exposure during visits to the rehabilitation outpatient department. Guardians of patients in poor condition, who were unable to visit the rehabilitation outpatient department, were interviewed to assess patients’ conditions.

### 2.4. Study outcomes

The primary outcomes of this study were neurocognitive outcomes at 1 month post-CO exposure in patients in the one-session group, compared with that in patients in the multiple-session group.

The secondary outcomes were neurocognitive outcomes at 1 month post-CO exposure in patients in the one-session group compared with that in patients in the multiple-session group in the mild (non-invasive mechanical ventilation) versus severe (invasive mechanical ventilation) groups.

### 2.5. Statistical analysis

Propensity score matching using the nearest neighbor method was performed to reduce selection bias in the observational study and control for confounders. The matching ratio was 1:2, and was based on age, sex, and statistically significant variables. Considering the study results on the optimal caliper width, the caliper width was set to 0.2 (14). The matching balance was evaluated as acceptable if the absolute value of the standardized mean difference was within 0.25 (15).

Data were reported as median (interquartile range) for continuous variables and frequency (percentages) for categorical variables. Normality was tested using the Shapiro–Wilks test. Differences in the number of HBO_2_ sessions were compared using the Mann–Whitney U test, for continuous variables, and the Chi-square test or Fisher’s exact test, for categorical variables. All statistical analyses were performed using SAS v9.4 (SAS Institute, Cary, North Carolina, US) and R v4.2.1 (R Core Team, Vienna, Austria). All statistical significance was confirmed at *p* < 0.05.

### 2.6. Ethics statement

The study was approved by Wonju Severance Christian Hospital’s institutional review board (approval number: CR322003) and was conducted according to the principles of the Declaration of Helsinki. Data from January 2006 to July 2020 were obtained from an existing prospective patient registry; the requirement for informed consent was waived because the analysis was retrospective, using prospectively collected registry data. The consecutive data after August 2020 were collected prospectively with informed consent (individual participants or legal guardians) for the “CARE CO cohort.”

## 3. Results

### 3.1. Study population characteristics

**Figure 1:**
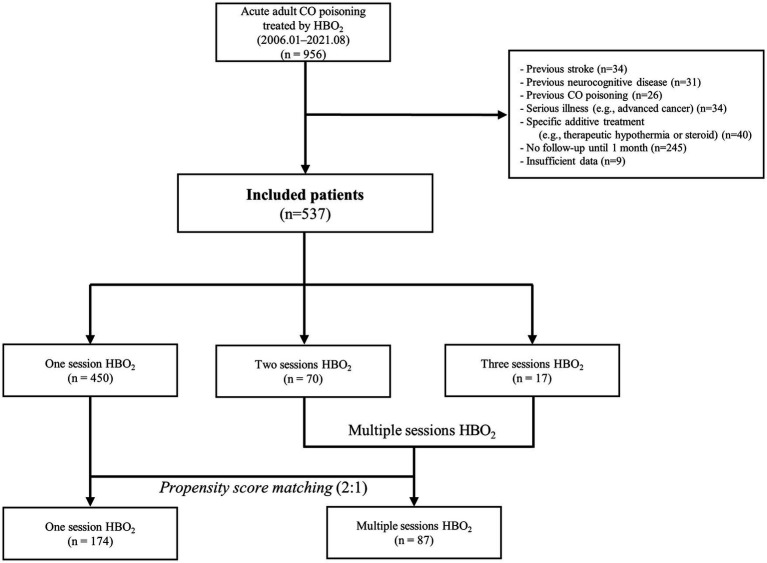
Study flow-chart. CO, carbon monoxide; HBO_2_, hyperbaric oxygen therapy.

Among the 956 patients (ages 16 years-70 years) with acute CO poisoning who were administered HBO_2_ therapy within 24 h after rescue from CO exposure, 537 patients presented in the final cohort were included (Figure 1).

The median age of the total cohort was 45 years (male, 66.3%). The most common source of CO was charcoal (74.5%). The median GCS score at the scene or ED was 15. The most common comorbidity was hypertension (14.5%). The median CO exposure time and time from rescue to first HBO_2_ were 3 h and 5.2 h in the total cohort, respectively. According to severity, the number of invasive mechanical ventilation and non-invasive mechanical ventilation patients was 39 (7.3%) and 498 (92.7%), respectively.

According to GDS, 34 patients (6.7%) had poor GDS scores before PSM. Three patients with GDS stages 4, 5, and 6 had neurological symptoms (motor weakness, speech disturbance, or peripheral neuropathy). Therefore, the neurocognitive outcome group classified by GDS combined with neurological impairment was not different from the outcome group categorized by GDS alone. Among the total patients, 450 (83.8%), 70 (13.0%), and 17 patients (3.2%) comprised the one-, two-, and three-session groups, respectively (Table 1). Overall, 10 patients died within 1 month after CO poisoning while under hospitalization due to uncontrolled metabolic acidosis, acute kidney injury, or profound shock.

**Table 1:** Baseline characteristics in the total cohort.

Variables	Total (*n* = 537)	Before PSM				After PSM	
		One session (*n* = 450)	Multiple sessions (*n* = 87)	Value of *p*	One session (*n* = 174)	Multiple sessions (*n* = 87)	Value of *p*
Age (years)	45 (35–56)	45 (35–56)	44 (35–55)	0.718	45 (35–55)	44 (35–55)	0.874
Sex (male)	356 (66.3)	292 (64.9)	64 (73.6)	0.117	123 (70.7)	64 (73.6)	0.627
Intentionality	196 (36.5)	162 (36)	34 (39.1)	0.585	79 (45.4)	34 (39.1)	0.331
Source							
Charcoal	400 (74.5)	331 (73.6)	69 (79.3)	0.091	102 (92.7)	52 (94.6)	0.536
Gas and oil	93 (17.3)	77 (17.1)	16 (18.4)		29 (16.7)	16 (18.4)	
Fire	44 (8.2)	42 (9.3)	2 (2.3)		9 (5.2)	2 (2.3)	
Drug co-ingestion	28 (5.2)	22 (4.9)	6 (6.9)	0.431	9 (5.2)	6 (6.9)	0.573
GCS score	15 (12–15)	15 (12–15)	15 (12–15)	<0.001	15 (12–15)	15 (12–15)	0.809
*Co-morbidities*							
Diabetes mellitus	44 (8.2)	36 (8)	8 (9.2)	0.710	19 (10.9)	8 (9.2)	0.666
Hypertension	78 (14.5)	66 (14.7)	12 (13.8)	0.832	24 (13.8)	12 (13.8)	1.000
Cardiovascular disease	15 (2.8)	11 (2.4)	4 (4.6)	0.281	3 (1.7)	4 (4.6)	0.227
Psychiatric disease	63 (11.7)	54 (12)	9 (10.3)	0.661	21 (12.1)	9 (10.3)	0.681
Alcohol co-ingestion	40 (7.4)	28 (6.2)	12 (13.8)	0.014	18 (10.3)	12 (13.8)	0.410
Current smoker	236 (43.9)	190 (42.2)	46 (52.9)	0.067	81 (46.6)	46 (52.9)	0.335
*Symptoms and sign at the ED*							
Loss of consciousness	273 (50.8)	214 (47.6)	59 (67.8)	<0.001	122 (70.1)	59 (67.8)	0.704
Shock	3 (0.6)	2 (0.4)	1 (1.2)	0.412	0 (0)	1 (1.2)	0.333
Seizure	6 (1.1)	5 (1.1)	1 (1.2)	1.000	3 (1.7)	1 (1.2)	0.397
CO exposure time (h)	3 (1–8)	3 (1–8)	5 (2–9)	0.007	4 (1–8)	5 (2–9)	0.367
Time from rescue to HBO (h)	5.2 (3.4–8.2)	5.4 (3.5–8.5)	4.6 (3.3–6.5)	0.019	4.5 (2.8–7)	4.6 (3.3–6.5)	0.660
*Laboratory findings*							
CO-Hb (%)	19.8 (8.3–30.4)	18.5 (8–30)	23.2 (11.1–32.3)	0.041	23.8 (12.4–35.3)	23.2 (11.1–32.3)	0.693
Bicarbonate (mmol/L)	21.5 (19.4–23.2)	21.6 (19.4–23.1)	21.3 (19.2–23.6)	0.701	21.2 (17.8–22.8)	21.3 (19.2–23.6)	0.092
Lactate (mmol/L)	2 (1.3–3.2)	2 (1.3–3.1)	2.2 (1.3–3.6)	0.158	2.4 (1.5–3.8)	2.2 (1.3–3.6)	0.640
Creatinine (mg/dL)	0.8 (0.6–1)	0.8 (0.6–1)	0.8 (0.7–1)	0.254	0.8 (0.7–1)	0.8 (0.7–1)	0.954
Creatine kinase (U/L)	129 (90–238)	129 (86–238)	131 (94–244)	0.369	136 (92–365)	131 (94–244)	0.804
Troponin I (ng/mL)	0 (0–0.1)	0.015 (0.015–0.060)	0.015 (0.006–0.241)	0.738	0.015 (0.015–0.139)	0.015 (0.006–0.241)	0.132
Invasive mechanical ventilation	39 (7.3)	32 (7.1)	7 (8.1)	0.758	18 (10.3)	7 (8.1)	0.552

Data are expressed as frequency (percentage) and median (interquartile range).

Two sessions = 70, Three sessions = 17 (after PSM).

PSM, propensity score matching; HBO_2_, hyperbaric oxygen therapy; GCS, Glasgow coma scale; ED, emergency department; CO, carbon monoxide; CO-Hb, carboxyhemoglobin.

### 3.2. Main results for neurocognitive outcomes

**Figure 2:**
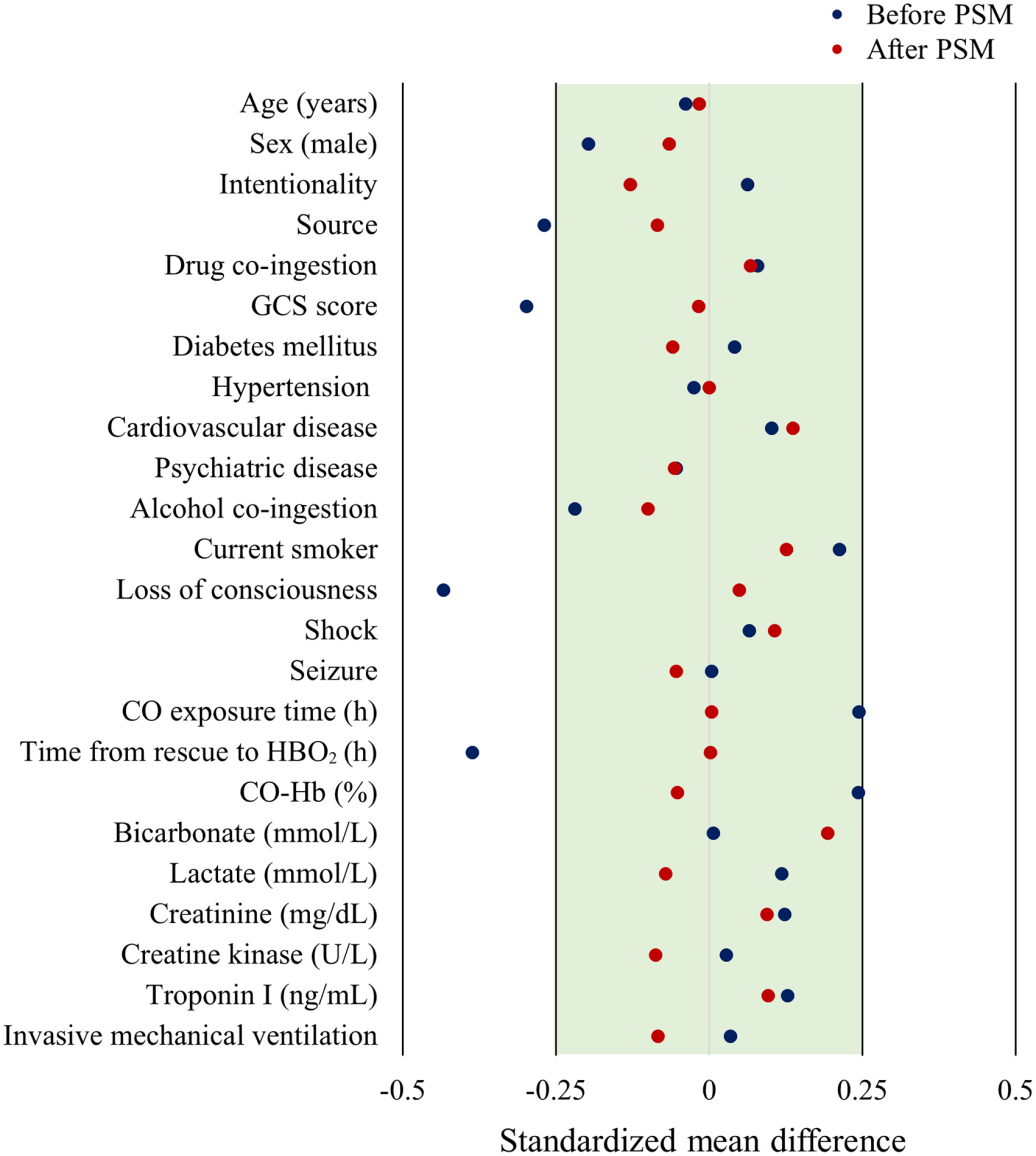
Forest plot of standardized mean difference based on before and after matching. PSM, propensity score matching; GCS, Glasgow coma scale; CO, carbon monoxide; HBO_2_, hyperbaric oxygen therapy; CO-Hb, carboxyhemoglobin.

**Figure 3:**
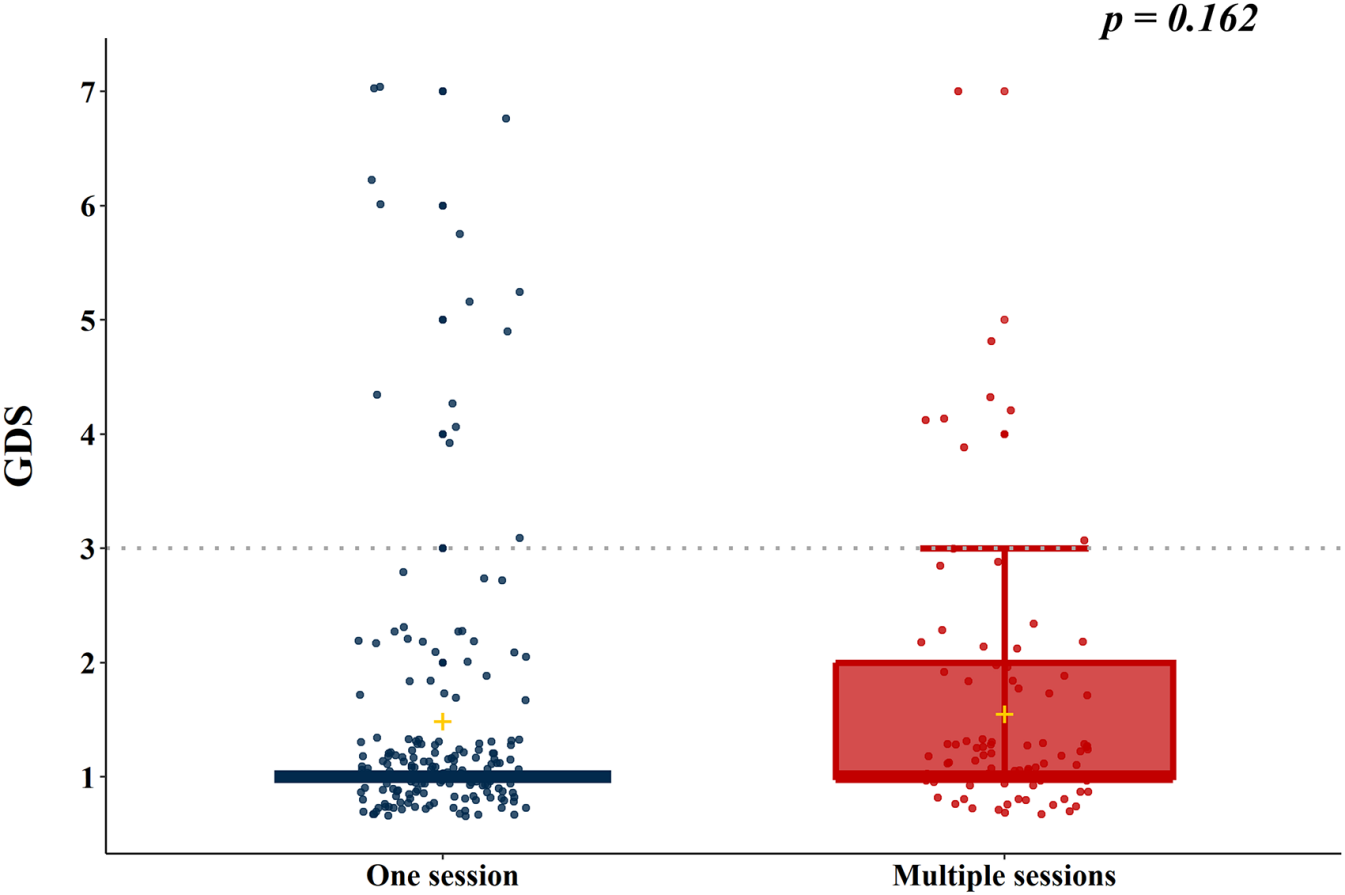
Neurocognitive outcome according to the number of hyperbaric oxygen therapy sessions in the total cohort. GDS, Global deterioration scale.

According to the results of the analysis of the baseline characteristics of patients, based on HBO_2_ sessions, there were significant differences in alcohol co-ingestion, loss of consciousness, GCS score, CO exposure time, time to rescue to HBO_2_, and CO-Hb level between the one-session and multiple-session groups (Table 1). After PSM using the covariates (age, sex, alcohol co-ingestion, loss of consciousness, GCS score, CO exposure time, time to rescue to HBO_2_, and CO-Hb level), no characteristic showed a statistically significant difference between the one-session and multiple-session groups. The matching balance was confirmed based on the absolute value of the standardized mean difference within 0.25 (Figure 2). In the entire cohort, there was no significant difference between the one-session group and the multiple-session group in terms of neurocognitive outcome (Table 2; Figure 3). In a subgroup analysis based on invasive mechanical ventilation, out of all matched patients, there were no significant differences between the one-session group and the multiple-session group (Tables 2-4).

**Table 2:** Neurocognitive outcomes.

Total cohort				
Variables	Total (*n* = 261)	After PSM		Value of *p*
		One session (*n* = 174)	Multiple sessions (*n* = 87)	
GDS	1.0 (1.0–1.0)	1.0 (1.0–1.0)	1.0 (1.0–1.0)	0.162
GDS category				0.869
Favorable (GDS 1–3)	241 (92.3)	161 (92.5)	80 (92)	
Poor (GDS 4–7)	20 (7.7)	13 (7.5)	7 (8.1)	
GDS category combined neurological impairment				
Favorable (GDS 1–3)	241 (92.3)	161 (92.5)	80 (92)	0.869
Poor (GDS 4–7)	20 (7.7)	13 (7.5)	7 (8.1)	
**Non-invasive mechanical ventilation cohort**				
**Variables**	**Total (*n* = 236)**	**After PSM**		**Value of *p***
		**One session (*n* = 156)**	**Multiple sessions (*n* = 80)**	
GDS category combined neurological impairment				
Favorable (GDS 1–3)	220 (93.2)	147 (94.2)	73 (91.3)	0.389
Poor (GDS 4–7)	16 (6.8)	9 (5.8)	7 (8.8)	
**Invasive mechanical ventilation cohort**				
**Variables**	**Total (*n* = 25)**	**After PSM**		**Value of *p***
		**One session (*n* = 18)**	**Multiple sessions (*n* = 7)**	
GDS category combined neurological impairment				
Favorable	21 (84)	14 (77.8)	7 (100)	0.295
Poor	4 (16)	4 (22.2)	0 (0)	

Data are expressed as frequency (percentage) and median (interquartile range).

Total cohort: two sessions = 70, three sessions = 17, Non-invasive mechanical ventilation cohort: two sessions = 67, three sessions = 13, Invasive mechanical ventilation cohort: two sessions = 3, three sessions = 4

PSM, propensity score matching; GDS, Global Deterioration Scale.

We evaluated whether the 1 month GDS score changed 6 months after CO poisoning in the total cohort (526 patients were followed up for up to 6 months). In 486 (92.4%) patients, the GDS stage remained unchanged; however, it improved in 38 (7.2%) and worsened in 2 (0.4%) patients. There was no significant difference in neurocognitive outcomes according to the number of HBO_2_ sessions (*p* = 0.179) (Supplementary Table 2).

## 4. Discussion

In this study, there was no significant difference in the neurocognitive sequelae at 1 month according to the number of HBO_2_ sessions performed within 24 h. In the 2018 annual scientific meeting of the Undersea and Hyperbaric Medical Society, Weaver et al. presented (unpublished) the results of a randomized trial comparing one and three HBO_2_ sessions for acute CO poisoning (16). They included English-speaking patients with accidental CO poisoning who arrived at the hospital within 24 h of exposure and were not intubated. There was no difference in the rate of neuropsychological sequelae in those who received 3 HBO_2_ sessions and those who received 1 HBO_2_ session and two sham sessions (6 weeks and 6 months after CO poisoning). For patients meeting the inclusion criteria stipulated in this study, one HBO_2_ treatment may be sufficient. However, they did not publish this study and included neither intentional poisoning nor severe CO poisoning (i.e., invasive mechanical ventilation patients). In our subgroup analysis according to severity, there was no significant difference in terms of neurocognitive sequelae at 1 month between the number of HBO_2_ sessions. This implies that regardless of severity, one session of HBO_2_ may be reasonable. Fujita et al. concluded that multiple HBO_2_ sessions were associated with the incidence of DNS (17). Although they conducted a prospective multicenter study, they used different HBO_2_ treatment protocols for each institution. In addition, among the 165 patients treated with HBO_2_ within the first 24 h, only 79 patients (47.9%) received the first HBO_2_ session with more than 2.8 ATA that could develop the therapeutic effect of HBO_2_ (18, 19) (see Tables 3, 4).

**Table 3:** Baseline characteristics in the non-invasive mechanical ventilation cohort.

Variables	Total (*n* = 236)	One session (*n* = 156)	Multiple sessions (*n* = 80)	
Age (years)	45.5 (35–55.5)	45.5 (35–55)	45.5 (35–56.5)	0.727
Sex (male)	168 (71.2)	109 (69.9)	59 (73.8)	0.534
Intentionality	95 (40.3)	66 (42.3)	29 (36.3)	0.369
Source				
Charcoal	187 (79.2)	123 (78.9)	64 (80)	1.000
Gas and oil	42 (17.8)	28 (18)	14 (17.5)	
Fire	7 (3)	5 (3.2)	2 (2.5)	
Drug co-ingestion	13 (5.5)	8 (5.1)	5 (6.3)	0.767
GCS score	15 (12–15)	15 (12–15)	15 (12–15)	0.576
*Co-morbidities*				
Diabetes mellitus	22 (9.3)	14 (9)	8 (10)	0.798
Hypertension	35 (14.8)	23 (14.7)	12 (15)	0.958
Cardiovascular disease	7 (3)	3 (1.9)	4 (5)	0.231
Psychiatric disease	25 (10.6)	18 (11.5)	7 (8.8)	0.510
Alcohol co-ingestion	28 (11.9)	16 (10.3)	12 (15)	0.286
Current smoker	114 (48.3)	72 (46.2)	42 (52.5)	0.356
*Symptoms and sign at the ED*				
Loss of consciousness	156 (66.1)	104 (66.7)	52 (65)	0.798
Shock	0 (0.0)	0 (0.0)	0 (0.0)	–
Seizure	3 (1.3)	3 (1.9)	0 (0)	0.553
CO exposure time (h)	4 (1.2–8)	3.8 (1–8)	5 (2–9)	0.378
Time from rescue to HBO_2_ (h)	4.3 (2.8–6.5)	4.3 (2.8–6.3)	4.4 (3.3–6.5)	0.360
*Laboratory findings*				
CO-Hb (%)	22.4 (10.6–32.4)	22.7 (10.8–34.4)	22.3 (9.4–31.6)	0.538
Bicarbonate (mmol/L)	21.4 (18.9–23.1)	21.4 (17.9–22.9)	21.4 (19.5–23.6)	0.154
Lactate (mmol/L)	2.3 (1.4–3.4)	2.3 (1.4–3.4)	2.2 (1.4–3.4)	0.890
Creatinine (mg/dL)	0.8 (0.7–1)	0.8 (0.7–1)	0.8 (0.7–1)	0.895
Creatine kinase (U/L)	131 (91.5–250.5)	132.5 (91–331.5)	130 (93.5–226.5)	0.834
Troponin I (ng/mL)	0.015 (0.015–0.112)	0.015 (0.015–0.101)	0.015 (0.006–0.142)	0.167

Data are expressed as frequency (percentage) and median (interquartile range).

Two sessions = 67, Three sessions = 13.

HBO_2_, hyperbaric oxygen therapy; GCS, Glasgow coma scale; ED, emergency department; CO, carbon monoxide; CO-Hb, carboxyhemoglobin.

**Table 4:** Baseline characteristics in the invasive mechanical ventilation cohort.

Variables	Total (*n* = 25)	One session (*n* = 18)	Multiple sessions (*n* = 7)	
Age (years)	38 (36–49)	43.5 (37–55)	36 (32–38)	0.057
Sex (male)	19 (76)	14 (77.8)	5 (71.4)	1.000
Intentionality	18 (72)	13 (72.2)	5 (71.4)	1.000
Source				
Charcoal	18 (72)	13 (72.2)	5 (71.4)	0.152
Gas and oil	3 (12)	1 (5.6)	2 (28.6)	
Fire	4 (16)	4 (22.2)	0 (0)	
Drug co-ingestion	2 (8)	1 (5.6)	1 (14.3)	0.490
GCS score	8 (5–8)	8 (8–8)	5 (5–8)	0.115
Co-morbidities				
Diabetes mellitus	5 (20)	5 (27.8)	0 (0)	0.274
Hypertension	1 (4)	1 (5.6)	0 (0)	1.000
Cardiovascular disease	0 (0.0)	0 (0.0)	0 (0.0)	–
Psychiatric disease	5 (20)	3 (16.7)	2 (28.6)	0.597
Alcohol co-ingestion	2 (8)	2 (11.1)	0 (0)	1.000
Current smoker	13 (52)	9 (50)	4 (57.1)	1.000
Symptoms and sign at the ED				
Loss of consciousness	25 (100)	18 (100)	7 (100)	–
Shock	1 (4)	0 (0)	1 (14.3)	0.280
Seizure	1 (4)	0 (0)	1 (14.3)	0.280
CO exposure time (h)	4 (1–8)	6.5 (0.5–8)	3 (1.3–8)	0.903
Time from rescue to HBO (h)	7.8 (4.6–14)	8.5 (4.9–14.5)	4.7 (3–13.4)	0.358
Laboratory findings				
CO-Hb (%)	40.3 (32.6–51.7)	35.3 (24.6–49.4)	51.2 (37.4–59.7)	0.109
Bicarbonate (mmol/L)	18.7 (15.3–21.8)	18.3 (15.3–21.4)	19.8 (12.1–24.3)	0.654
Lactate (mmol/L)	4.1 (2–5.8)	3.9 (2.1–5.8)	4.1 (1.3–6.5)	0.698
Creatinine (mg/dL)	0.9 (0.7–1.3)	0.9 (0.7–1.3)	0.8 (0.7–1.7)	0.765
Creatine kinase (U/L)	250 (127–1,148)	286 (108–529)	250 (127–2,506)	0.905
Troponin I (ng/mL)	0.408 (0.042–2.100)	0.358 (0.042–2.182)	0.492 (0.010–2.100)	0.928

Data are expressed as frequency (percentage) and median (interquartile range).

Two sessions = 3, Three sessions = 4.

HBO_2_, hyperbaric oxygen therapy; GCS, Glasgow coma scale; ED, emergency department; CO, carbon monoxide; CO-Hb, carboxyhemoglobin.

In previous RCTs, HBO_2_ with 2.8 or 3.0 ATA was more effective than NBO_2_ in patients with symptomatic CO poisoning, (4, 7, 8) particularly in patients treated within 6 h after CO exposure. In an RCT by Ducassé et al. (7), one session of HBO_2_ treatment was performed in patients with GCS >12 with an elapsed time between discovery and hospitalization of less than 2 h. In an RCT by Thom et al. (8), one HBO_2_ session was performed within 6 h in patients with no loss of consciousness or cardiac instability. Two RCTs (7, 8) showed that HBO_2_ was superior to NBO_2_ in reducing neurocognitive sequelae. In addition, in an RCT by Weaver et al. (4), HBO_2_ was given in three sessions in all patients, regardless of severity, and this treatment (mean exposure-to-treatment interval: 5.8 h) reduced neurocognitive sequelae incidence by half at 6 weeks, and from 25 to 18% at the 1 year evaluation, compared with no HBO_2_ treatment. It means that in existing RCTs, both one and three HBO_2_ sessions performed within 6 h worked more effectively than NBO_2_ in patients with acute CO poisoning. Our results, combined with the previous RCTs (4, 7, 8) suggest that the first HBO_2_ (2.8 or 3.0 ATA) session administered rapidly after CO exposure is the most important and effective. Meanwhile, some RCTs did not exhibit the therapeutic effect of HBO_2_ (6, 9, 10). However, in a study by Raphael et al. (6), an insufficient dose of HBO_2_ was used at 2.0 ATA, and nearly half of the study group was treated after more than 6 h post-exposure. Annane et al. (10) also used an insufficient dose of HBO_2_ (2.0 ATA). For the optimal effect of HBO_2_, adequate pressure is necessarily required (1). Although 2.8 ATA was used in the study by Scheinkestel et al. (9), the 1 month cognitive outcomes were not evaluated. Instead, the only cognitive outcomes provided were reported a few days after poisoning. In addition, three RCTs that showed no beneficial effects of HBO_2_ did not meet the CONSORT guidelines, unlike the RCT conducted by Weaver et al. (4).

The findings of this study can be understood in terms of the time frame of pathophysiology by CO poisoning and the HBO_2_ mechanism of action. Acute CO poisoning causes neutrophil degranulation by activating intravascular neutrophils through platelet–neutrophil aggregates (20). This process leads to oxidative stress, transformation of xanthine dehydrogenase to xanthine oxidase in endothelial cells, lipid peroxidation, and apoptosis by causing the release of myeloperoxidase, proteases, and reactive oxygen species (20, 21). Finally, these reactions develop an adaptive immunological response causing CO-mediated neurocognitive sequelae by the formation of chemically modified myelin basic protein (22). These CO-mediated inflammatory reactions proceed rapidly after CO exposure. The leukocyte adherence in the vascular endothelial cells that triggers these changes occurs early. In the first 90 min after CO poisoning in an animal model, xanthine dehydrogenase is converted to xanthine oxidase, and reactive oxygen species produced by xanthine oxidase result in lipid peroxidation, which is temporary, dissipating within 16 h (23).

In terms of the time frame of the HBO_2_ mechanism of action, exposure to 2.8 or 3.0 ATA HBO_2_ can inhibit leukocyte ß_2_-integrin function by S-nitrosylation and cell adherence to the cerebral microvasculature, inhibiting the sequential immunological reaction, as shown in both animal and human studies (18, 19). HBO_2_ temporarily inhibits adherence within 2 h after treatment (19). Additionally, HBO_2_ efficiently protects from neuronal apoptosis 3–5 h after CO poisoning (24). In rats treated with HBO_2_ 45 min after CO exposure, HBO_2_ prevented immune-mediated neurological damage through partial prevention of chemically modified myelin basic protein formation (25). Therefore, early HBO_2_ administration at 2.8 or 3.0 ATA is the most important because it may stop the process early, in turn preventing brain injury related to an adaptive immunological response.

There are a few limitations to this study. First, this was an observational, non-randomized study. From an analytical perspective, PSM was used to minimize bias due to the study design (26). Nevertheless, hidden bias may remain due to the effects of unmeasured confounding variables. However, this is the first study with a large sample size (>500 patients). Second, the number of patients who received three sessions of HBO_2_ in this study was small. Weaver et al. chose to provide three HBO_2_ sessions because a retrospective report suggested that the use of more than two treatments resulted in better outcomes than that of a single treatment (27). In addition, they provided the three sessions within a 24 h period because they anticipated that patients’ compliance would be better during a shorter period than a longer one. However, it is practically very difficult to perform three sessions at intervals of 6–12 h, all within 24 h at the clinical site. Third, we did not evaluate the effects of additional HBO_2_ after 24 h. To date, no study has evaluated additional HBO_2_ after 24 h. Therefore, we excluded patients with additional HBO_2_ sessions after 24 h. Fourth, although few RCTs have conducted more than 6 neurocognitive tests, usually equivalent to CO batteries (4, 8), we only evaluated outcomes with the GDS combined with neurological impairment. Our institute uses the GDS stage to evaluate neurocognitive prognosis in patients with CO poisoning because it has the advantage of recognizing neurocognitive functions, such as memory and concentration, as well as activities of daily living, through interviews. We have previously reported the GDS stage for the measurement of neurocognitive outcomes in a study related to CO poisoning (11, 13, 28). Fifth, a few patients were lost to follow-up due to their condition, distance from the hospital, or poor compliance. Sixth, charcoal was the most common CO source in this study. Gas and oil combustion, which are the main sources of CO poisoning in Western countries, were the CO sources in 93 patients of the total cohort. In the gas and oil combustion-exposed subpopulation, there was no significant difference according to the number of HBO_2_ sessions (*p =* 0.632) (Supplementary Table 3).

There were no differences in terms of the reduction in poor neurocognitive outcomes according to the number of HBO2 sessions administered within 24 h after CO poisoning. In addition, there was no difference according to severity in terms of neurocognitive outcomes. Therefore, one HBO2 treatment within 24 h may be reasonable.

## Data availability statement

The original contributions presented in the study are included in the article/Supplementary material, further inquiries can be directed to the corresponding author.

## Ethics statement

The studies involving human participants were reviewed and approved by Wonju Severance Christian Hospital’s institutional review board. Written informed consent to participate in this study was provided by the participants’ legal guardian/next of kin.

## Author contributions

YC conceived the study and designed the trial, obtained research funding, supervised the conduct of the study and data collection, and takes responsibility for the paper as a whole. JL provided statistical advice on study design and analyzed the data. JSL and YC drafted the manuscript, and all authors contributed substantially to its revision. All authors contributed to the article and approved the submitted version.

## Funding

This work was supported by the National Research Foundation of Korea, which is funded by the Korean government (Ministry of Science and Information and Communications Technology, grant no. NRF-2021R1A2C200492211).

## Conflict of interest

The authors declare that the research was conducted in the absence of any commercial or financial relationships that could be construed as a potential conflict of interest.

## Publisher’s note

All claims expressed in this article are solely those of the authors and do not necessarily represent those of their affiliated organizations, or those of the publisher, the editors and the reviewers. Any product that may be evaluated in this article, or claim that may be made by its manufacturer, is not guaranteed or endorsed by the publisher.

## Supplementary material

The Supplementary material for this article can be found online at: https://www.frontiersin.org/articles/10.3389/fmed.2023.1127978/full#supplementary-material
