# Supplementary material for: Association between number of hyperbaric oxygen therapy sessions and neurocognitive outcomes of acute carbon monoxide poisoning
Source: Front Med (Lausanne). 2023 Feb 14;10:1127978. doi: 10.3389/fmed.2023.1127978 (PMC10127102; doi:10.3389/fmed.2023.1127978)
Supplement: Supplementary file 1 [file Data_Sheet_1.docx]

Supplementary Material

Association between Number of Hyperbaric Oxygen Therapy Sessions and Neurocognitive Outcomes of Acute Carbon Monoxide Poisoning

Je Seop Lee^1,2^, Yong Sung Cha^1,2*^, Jihye Lim^3^

^1^ Department of Emergency Medicine, Yonsei University Wonju College of Medicine, Wonju, Republic of Korea

^2^Research Institute of Hyperbaric Medicine and Science, Yonsei University Wonju College of Medicine, Wonju, Republic of Korea

^3^Department of Biostatistics and Center of Biomedical Data Science, Yonsei University Wonju College of Medicine, Wonju, Republic of Korea

*** Correspondence:**Yong Sung Cha, MD, PhD
[emyscha@yonsei.ac.kr](mailto:emyscha@yonsei.ac.kr)

Supplementary methods

# Variable definitions

The patient was diagnosed with shock when a vasopressor was required for resuscitation. The carbon monoxide (CO) exposure duration obtained from patients’ guardians was defined as the estimated maximum duration of CO exposure measured from the time of normal consciousness to patient detection. The time from rescue to first hyperbaric oxygen therapy (HBO_2_) was defined as the time from rescue from the CO source to the start of the first HBO_2_ session.

# Global Deterioration Scale

The Global Deterioration Scale (GDS) is a validated, reliable instrument for describing the clinical progression of dementia.^1^ It is also used to determine the prognosis of patients with carbon monoxide (CO) poisoning^2-4^; and those with severe chronic obstructive pulmonary disease, Alzheimer’s disease, and vasculopathy-related dementia.^1,5-7^

Although the GDS score is not as diverse as a CO battery, it has the advantage of being able to identify neurocognitive functions, such as memory and concentration, as well as activities of daily living through interviews. Moreover, performance of multiple neurocognitive function tests may be difficult in patients with disease sequelae. The Short-Form General Health Survey-36, a commonly used testing tool, has a set of self-reported questions; however, it is limited in evaluating patients with severe neurological impairment as it requires an individual’s ability to understand and address the questions. Digit span, trail making, and clock drawing are good evaluation tools but require short-term memory and visuospatial functions. The GDS score can be used for all patients with CO poisoning, regardless of poisoning severity. The scale consists of 7 scores, with higher scores indicating greater severity.

**References**

1. Reisberg B, Ferris SH, de Leon MJ, et al. The Global Deterioration Scale for assessment of primary degenerative dementia. *Am J Psychiatry* 1982;139:1136-1139
2. Kim SJ, Thom SR, Kim H, et al. Effects of adjunctive therapeutic hypothermia combined with hyperbaric oxygen therapy in acute severe carbon monoxide poisoning. *Crit Care Med* 2020;48:e706-e714
3. Cho DH, Ko SM, Son JW, et al. Myocardial injury and fibrosis from acute carbon monoxide poisoning: a prospective observational study. *JACC Cardiovasc Imaging*. 2021;14:1758-1770
4. Lee Y, Cha YS, Kim SH, et al. Effect of hyperbaric oxygen therapy initiation time in acute carbon monoxide poisoning. *Crit Care Med*. 2021;49:e910-e919
5. Paul RH, Cohen RA, Moser DJ, et al. The global deterioration scale: Relationships to neuropsychological performance and activities of daily living in patients with vascular dementia. *J Geriatr Psychiatry Neurol* 2002;15:50-54
6. Eisdorfer C, Cohen D, Paveza GJ, et al. An empirical evaluation of the Global Deterioration Scale for staging Alzheimer's disease. *Am J Psychiatry* 1992; 149:190-194
7. Ozge C, Ozge A, Unal O. Cognitive and functional deterioration in patients with severe COPD. *Behav Neurol* 2006;17:121-130

Supplementary Table 1. Global Deterioration Scale

| Stage | Cognitive dysfunction | Clinical characteristics |
| --- | --- | --- |
| 1 | No cognitive decline | *Patients appear clinically normal.*  No complaints of memory deficits.  No evident memory deficit on clinical interview. |
| 2 | Very mild cognitive decline | *Patients complain of memory deficits.*  Most frequently, patients:  (a) forget where they have placed familiar objects;  (b) forget the name of someone they formerly knew well.  No objective evidence of memory deficit on clinical interview.  No objective deficits in employment or social situations.  Patients display appropriate concern about their symptoms. |
| 3 | Mild cognitive decline | *Earliest clear-cut deficits*.  Objective evidence of memory deficit was obtained only with an intensive interview conducted by a trained geriatric psychiatrist. Concentration deficit may be evident on clinical testing.  Patients may demonstrate a reduced ability to:  (a) remember names upon introduction to new people;  (b) retain information after reading a passage from a book.  Decreased performance manifests in demanding employment and social situations. Examples may include:  (a) coworkers becoming aware of the patient’s relatively poor performance;  (b) difficulties in finding words and names becoming evident to intimate acquaintances;  (c) losing or misplacing objects of value;  (d) getting lost when traveling to unfamiliar locations.  The subtlety of the clinical symptoms may be exacerbated by denial, which is often evident in these patients. Mild-to-moderate anxiety also accompanies the symptoms, typically when the patients are forced to cope with challenging employment and social demands that they find they can no longer negotiate. |
| 4 | Moderate cognitive decline | *Clear-cut deficits on careful clinical interview.*  Deficits manifest in many areas, such as:  (a) concentration deficit elicited in serial subtractions;  (b) decreased knowledge of current events and recent life events;  (c) upon careful questioning, patients may exhibit a deficit in memory of their personal history;  (d) decreased ability to travel alone and manage finances.  Patients can no longer perform complex tasks accurately and efficiently. However, certain abilities remain preserved, such as:  (a) orientation to time and people;  (b) familiar persons and faces can be distinguished from strangers;  (c) ability to travel to familiar locations.  Denial is often the dominant defense mechanism. The evident decline in the patients’ intellectual and cognitive capacities is too overwhelming with a loss for full conscious acceptance and recognition. A flattening of effect and withdrawal from previously challenging situations are observed. |
| 5 | Moderately severe cognitive decline | *Patients can no longer survive without some assistance.*  During interviews, patients are unable to recall a major relevant aspect of their current lives. Examples include:  (a) difficulty recalling their address or telephone number, names of close family members, such as grandchildren, or the name of the high school or university from which they graduated;  (b) some disorientation to time (date, day of the week, season) or location;  (c) well-educated patients may have difficulty counting backwards from 40 by 4s or from 20 by 2s.  Patients retain the knowledge of many major facts regarding themselves and others. They invariably know their own names and generally know their spouse and children’s names. They require no assistance with toileting and eating but may have some difficulty choosing the proper clothing to wear and may occasionally clothe themselves improperly (e.g., put their shoes on the wrong feet). |
| 6 | Severe cognitive decline | *Patients may occasionally forget the name of their spouse, on whom they depend entirely for survival.*  Patients are largely unaware of all recent events and experiences in their lives.  They retain some knowledge of their past, but this knowledge is very uncertain. They are generally unaware of their surroundings, the year, or the season and may have difficulty counting backward, and sometimes forward, from 10. Patients require substantial assistance with activities of daily living. These are quite variable and include:  (a) delusional behavior (e.g., patients may accuse their spouse of being an impostor, may talk to imaginary figures in the environment, or their own reflection in the mirror);  (b) obsessive symptoms (e.g., continual repetition of simple cleaning activities);  (c) anxiety symptoms, agitation, and previously nonexistent violent behavior;  (d) cognitive abulia (i.e., loss of willpower because they cannot carry a thought long enough to determine a purposeful course of action). |
| 7 | Very severe cognitive decline | *All verbal abilities are lost.*  Frequently there is no speech at all, and only grunting remains.  Patients have urinary incontinence and require assistance with toileting and eating. They lose psychomotor skills (e.g., the ability to walk). The brain appears no longer able to tell the body what to do. Generalized cortical and focal neurologic signs and symptoms are frequently present. |

Supplementary Table 2. Change in Global Deterioration Scale (GDS) at 1 month and 6 months

| Difference between  GDS 1 month and  GDS 6 months | Total | HBO_2_ sessions | | P-value |
| --- | --- | --- | --- | --- |
|  |  | One session (n=445) | Multiple sessions (n=81) |  |
| Improved | 38 (7.2) | 29 (6.5) | 9 (11.1) |  |
| No change | 486 (92.4) | 414 (93) | 72 (88.9) | 0.305 |
| Worsened | 2 (0.4) | 2 (0.5) | 0 (0.0) |  |

Data are expressed as a frequency (percentage)

P-value was calculated by Fisher’s exact test

Eleven patients were not administered GDS at 6 months

Two sessions = 65, Three sessions = 16

GDS = Global Deterioration Scale; HBO_2_ = hyperbaric oxygen therapy.

Supplementary Table 3. Baseline characteristics in gas and oil cohorts

| Variables | Total (n=45) | HBO_2_ sessions | | P‒value |
| --- | --- | --- | --- | --- |
|  |  | One session (n=29) | Multiple sessions (n=16) |  |
| Age (years) | 53 (36–60) | 53 (39–58) | 46.5 (30–61.5) | 0.850 |
| Sex (male) | 38 (84.4) | 22 (75.9) | 16 (100) | 0.040 |
| Intentionality | 2 (4.4) | 2 (6.9) | 0 (0) | 0.531 |
| Drug co-ingestion | 1 (2.2) | 0 (0) | 1 (6.3) | 0.356 |
| GCS score | 15 (12–15) | 15 (15–15) | 15 (10–15) | 0.333 |
| Co-morbidities |  |  |  |  |
| Diabetes mellitus | 4 (8.9) | 2 (6.9) | 2 (12.5) | 0.608 |
| Hypertension | 9 (20) | 6 (20.7) | 3 (18.8) | 1.000 |
| Cardiovascular disease | 2 (4.4) | 1 (3.5) | 1 (6.3) | 1.000 |
| Psychiatric disease | 1 (2.2) | 1 (3.5) | 0 (0) | 1.000 |
| Alcohol co-ingestion | 0 (0.0) | 0 (0.0) | 0 (0.0) | – |
| Current smoker | 25 (55.6) | 14 (48.3) | 11 (68.8) | 0.224 |
| Symptoms and sign at the ED |  |  |  |  |
| Loss of consciousness | 24 (53.3) | 16 (55.2) | 8 (50) | 0.739 |
| Shock | 0 (0.0) | 0 (0.0) | 0 (0.0) | – |
| Seizure | 0 (0.0) | 0 (0.0) | 0 (0.0) | – |
| CO exposure time (h) |  |  |  |  |
| Time from rescue to HBO_2_ (h) | 5 (1–9) | 7 (1–16) | 5 (2.5–7) | 0.547 |
| Laboratory findings | 4.7 (3.3–7.7) | 4.4 (2.5–7.7) | 5.2 (3.5–9.1) | 0.391 |
| CO-Hb (%) | 25.2 (14.7–37) | 24 (14.7–37) | 30.3 (11–37.2) | 0.916 |
| Bicarbonate (mmol/L) | 22.1 (19.7–23.5) | 21.8 (20.1–23.4) | 22.9 (19.6–24.3) | 0.297 |
| Lactate (mmol/L) | 1.8 (1.1–3.9) | 2 (1.3–4.1) | 1.5 (1–3) | 0.354 |
| Creatinine (mg/dL) | 0.8 (0.7–1) | 0.8 (0.7–1) | 0.9 (0.8–1.1) | 0.044 |
| Creatine kinase (U/L) | 158 (104–203) | 149 (93–203) | 190 (132.5–268.5) | 0.211 |
| Troponin I (ng/mL) | 0.015 (0.006–0.04) | 0.015 (0.009–0.021) | 0.016 (0.003–0.593) | 0.634 |
| Invasive mechanical ventilation | 3 (6.7) | 1 (3.5) | 2 (12.5) | 0.285 |
| GDS category combined neurological impairment |  |  |  | 1.000 |
| Favorable (GDS 1–3) | 43 (95.6) | 28 (96.6) | 15 (93.8) |  |
| Poor (GDS 4–7) | 2 (4.4) | 1 (3.5) | 1 (6.3) |  |

Data are expressed as a frequency (percentage) for categorical variables and median (interquartile range) for continuous variables.

Two sessions = 10, Three sessions = 6

HBO_2_ = hyperbaric oxygen therapy; GCS = Glasgow Coma Scale; ED = emergency department; CO = carbon monoxide; CO-Hb = carboxyhemoglobin; GDS = Global Deterioration Scale.
